# Computerized Physician Order Entry Systems: The Coming of Age for Outpatient Medicine

**DOI:** 10.1371/journal.pmed.0020290

**Published:** 2005-09-27

**Authors:** Robert L Davis

## Abstract

Davis discusses a new study in *PLoS Medicine* that evaluates the effectiveness of automated computerized alerts at reducing prescription errors in a primary care setting.

In this month's issue of *PLoS Medicine*, Steele et al. address a timely question in clinical medicine today—how best to use the potential of computers to change and improve physicians' prescribing behavior [1]. In evaluating the effectiveness of automated computerized alerts in a primary-care setting, the authors examined a system that had been developed to reduce the chances of a physician prescribing a medication that would lead to any of the following five different drug-induced adverse events: hypokalemia, hyperkalemia, nephrotoxicity, thrombocytopenia, or hepatotoxicity. Specific rules triggered alerts that were delivered via computer to the prescribing physician whenever there existed a potential for one or more of these five adverse events. These alerts presented suggestions for the physician during the prescription-writing process; faced with an alert, a physician could elect to proceed, revise, or stop a medication order, or order additional laboratory tests.

## The Study Design

Steele and colleagues used a “before and after” design to assess the impact of the intervention on physicians' prescribing behavior over a nine-month study period, during which 19,076 patients were seen. In the “before” phase, which lasted 17 weeks, the rules were switched on in the background without displaying any message to the providers. Since the rules were processing in the background, the providers did not receive any alerts recommending changes to their orders. Physicians' baseline ordering behavior was then compared to their ordering behavior in the “after” phase, during which alerts were presented to the provider. Adverse drug events were also assessed before and after the intervention by doing a random sample of chart reviews. [Fig pmed-0020290-g001]

**Figure pmed-0020290-g001:**
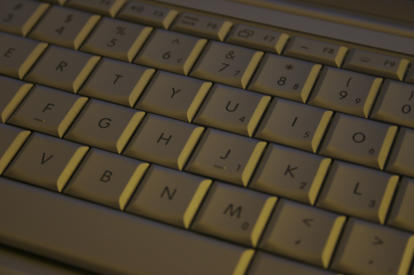
Can computers help to reduce prescribing errors?

## Key Findings

During the six months after introduction of the intervention, almost 50% of all the medications prescribed triggered an assessment by the system. Alerts were presented to providers over 1,000 times (for about 6% of all the completed prescriptions). These alerts indicated a possible “lab value–prescription mismatch,” for instance, a lab test that was abnormal or absent, and the physician needed to account for this. After the intervention, there was a significant increase in the percentage of time that physicians ordered a rule-associated laboratory test (39% at baseline versus 51% post-intervention), particularly if the relevant laboratory value was not present in the data files. There was also a significant increase in the percentage of cases in which the provider stopped the ordering process and did not complete the medication order when an alert for an abnormal rule-associated laboratory result was displayed (5.6% versus 10.9%). There was also a nonsignificant decrease in the percentage of patients who had potential for an adverse drug event (10.3% versus 4.3%; *p* = 0.23).

## Can Computers Help to Change Physicians' Behavior?

It has been quite difficult to work out how to develop interventions (using, for example, educational interventions or local experts and opinion leaders) that change physicians' behavior in ways that are generalizable, cost-efficient, clinically important, and persistent. Many of these same challenges exist with computers, where it has also been difficult to demonstrate important, clinically relevant, and lasting benefits from systems such as computerized alerts. The study by Steele et al. is important, as it demonstrates an ability to improve safety in prescribed medications, although it is equally important to acknowledge that this study showed little or no direct clinical benefit to patients.

This study adds further evidence to suggest that computerized systems for clinical medicine may be valuable. The study is a necessary step toward demonstrating the benefit of computerized physician order entry (CPOE) systems in the outpatient setting. To date, assessments of CPOE systems in the outpatient setting have been depressingly rare, with most studies of CPOE systems having been performed on patients in hospitals or in emergency departments. Since these clinical locations have had to deal with more complicated situations and more critically ill patients, it is not surprising that the early attempts by hospitals to automate their clinical information systems concentrated on such settings. There is now substantial evidence showing the benefit of different types of CPOE systems for hospitalized patients [2], but unfortunately there is little evidence for its effectiveness in the outpatient setting, where most prescriptions are written.

Studies of CPOE systems among critically ill hospitalized patients are not generalizable to the outpatient setting: not only do the types of prescriptions differ (typically oral for outpatients, while often intravenous or intramuscular for inpatients), but the patient issues differ as well. CPOE systems in the outpatient setting have to be geared mostly toward chronically ill patients with multiple needs centered on long-term management, whereas inpatient systems have to function for issues of acute illness in critically ill hospitalized patients. Hence, CPOE-based improvements in one setting cannot be assumed to extend to the other, since the conditions they deal with and the types of demands on the systems are so different.

## Limitations of the Study

Although Steele and colleagues' study showed a favorable effect for the use of computerized alert systems, there were some limitations. Although there was an increase in laboratory tests ordered when previous test values were either abnormal or absent, the study was not powered—as the authors themselves note—to demonstrate a reduction in adverse drug events. Use of surrogate outcomes (such as changes in lab ordering behavior) is a well-accepted practice in studies where adverse outcomes are very rare and where such surrogates are linked to the occurrence of adverse outcomes. Nevertheless, before some clinics or practice groups decide to invest in expensive systems that will impact the day-to-day activities of many health-care providers, more studies of CPOE systems demonstrating not only a change in physician behavior but a real reduction in adverse events will likely be needed.

Also, because this study was not a randomized clinical trial, it is necessary to consider other reasons for the findings. In any “before and after” study such as this one, improvements in prescribing patterns over time may occur, regardless of whether or not the intervention was effective. External factors (such as statewide initiatives to improve prescription practices) or internal factors (such as increased attention by individual clinic physicians to the problem of drug–lab interactions) may lead to the erroneous observation that the intervention was effective when, in fact, improvements were a result of these other influences.

Assessments of CPOE systems in the outpatient setting have been depressingly rare.

A randomized clinical trial would have avoided the problems of trends over time and, if done correctly, could have reduced contamination by other factors. However, such trials are often lengthy and expensive. In comparison, many additions to CPOE systems are incremental, and interest in any particular change is often time-limited. Additionally, in many situations, CPOE systems are initiated by clinic-affiliated quality improvement departments, which consider numerous factors when deciding to purchase or develop such systems. In these cases, there may be little motivation to study individual aspects of such a system, much less undertake a costly randomized trial. Hence, for many changes, it will likely not be feasible to evaluate each one by way of randomized clinical trials.

## Conclusion

Regardless of their limitations, observational studies such as the one by Steele et al. that compare nonrandomized physicians' behavior before interventions to behavior after interventions, and that depend largely on surrogate outcomes, provide powerful evidence on the effectiveness of computerized systems to reduce medication errors and improve patient safety. Similar studies will likely continue to be an important means used to evaluate many future developments in CPOE technology.

## Abbreviation

CPOE: computerized physician order entry
